# Prevalence of enteric parasitic infections among people living with HIV in Abeokuta, Nigeria

**DOI:** 10.11604/pamj.2018.30.66.13160

**Published:** 2018-05-28

**Authors:** John Kehinde Amoo, Akeem Abiodun Akindele, Abimbola Oladipupo Joseph Amoo, Akinwale Michael Efunshile, Taiwo Adetola Ojurongbe, Samuel Adetona Fayemiwo, Bolaji Nun Thomas, Olusola Ojurongbe

**Affiliations:** 1 Department of Medical Microbiology & Parasitology, Olabisi Onabanjo University, Ago-Iwoye, Nigeria; 2 Department of Medical Microbiology & Parasitology, Ladoke Akintola University of Technology, Osogbo, Nigeria; 3 Department of Medical Microbiology, Ebonyi State University, Abakaliki, Nigeria; 4 Department of Mathematical and Physical Sciences, Osun State University, Osogbo, Nigeria; 5 Department of Medical Microbiology, College of Medicine, University of Ibadan, Ibadan, Nigeria; 6 Department of Biomedical Sciences, College of Health Sciences and Technology, Rochester Institute of Technology, Rochester NY, USA

**Keywords:** Enteric pathogens, HIV/AIDS, CD4+ count, diarrhea

## Abstract

**Introduction:**

Enteric parasitic infections have been increasingly recognized as etiology of life-threatening chronic diarrhea in PLWHA in sub-Saharan Africa. This study investigated the prevalence and burden of intestinal parasitic infection among PLWHA in Abeokuta, southwest Nigeria.

**Methods:**

Freshly passed stool samples were collected from PLWHA. Detection of *Cryptosporidium spp* and *Microsporidium* spp was carried out with Kinyoun's stain and Weber's Chromotrope-based stain respectively. Investigation of other intestinal parasites was done using the direct saline preparation and formol-ether concentration methods. CD4+ T cell count was measured using Partec flow cytometry technique

**Results:**

A total of 231 (males: females 96:135; mean age 31.81±11.40 years) PLWHA were recruited into the study, among whom 84 (36.4%) were infected with at least one intestinal parasites. Fifty two (22.5%) individuals were positive for *Cryptosporidium spp* and a significant association between *Cryptosporidium spp*and diarrhea was observed (p=0.006). Seven (3.0%) were positive for *Microsporidium* spp. Helminths recovered included *Ascaris lumbricoides* (20.8%), hookworm (6.5%), *Strongyloides stercoralis* (4.3%), *Trichuris trichiura* (5.6%) and *Taenia spp.* (5.6%). *Cryptosporidium spp*, *Microsporidium* spp and *S. stercoralis* were significantly associated with CD4+ count ≥ 200 cells/mm^3^ (p<0.05). *Cryptosporidium spp*and A. lumbricoides were significantly observed among patients that are anti-retroviral therapy (ART) naive.

**Conclusion:**

High prevalence of opportunistic parasitic infection was significantly correlated with diarrhea, low CD4+ count and ART naïve individuals in the study. These findings re-emphasize the need for early diagnosis of opportunistic parasites and appropriate intervention among PLWHA.

## Introduction

Enteric parasitic infections are important and common features of Human Immunodeficiency Virus infection (HIV), causing significant morbidity and responsible for about 80% of Acquired Immune Deficiency Syndrome (AIDS)-related deaths [1,2]. Co-infections of HIV and opportunistic parasites, including intestinal protozoa and helminths, are of concern in resource-poor settings where the health status of the population is generally poor and these opportunistic parasites very common [3]. The resultant effect of such parasitic infections include chronic diarrhea, weight loss, and malnutrition, which has been associated with death among AIDS patients [4]. HIV has the capacity to circumvent and weaken human immune system providing the impetus for increased infection with parasites such as *Cryptosporidium spp*., *Microsporidium* spp, *Giardia intestinalis*, and *Strongyloides (S) stercoralis* [4].

*Cryptosporidium spp, Microsporidia* and other coccidian parasites have emerged as significant causes of persistent diarrhea in People living with HIV/AIDS (PLWHA) [5]. These pathogens have been recognized as worldwide causes of diarrhea in all age groups, yet their most significant impact have been felt among individuals with weakened immune systems, especially PLWHA and organ transplant recipients [6]. In immunocompromised individuals, diarrheal infections goes beyond the inconvenience of frequent watery stool but may result in severe and potentially life-threatening dehydration, electrolyte loss and malnutrition, and eventually death [7]. Transmission of *Cryptosporidium* is mainly through the fecal-oral route in contaminated water and food, as well as through person-to-person spread and contact with infected animals [8]. Microsporidiosis, caused by *Microsporidia*, another important opportunistic pathogen causing significant morbidity in PLWHA [9], is also associated with life-threatening chronic diarrhea and systemic disease [10] and are caused by members of *Enterocytozoon* and *Encephalitozoon* genera [11]. The route of transmission is usually by ingestion of the spores, including evidence of spore inhalation or rectal transmission [12].

Nigeria has the highest number of PLWHA (3.2 million) after South Africa (6.8 million) [13]. HIV causes progressive depletion of the CD4+ T cells, leading to life-threatening opportunistic infections during the natural course of the disease [14]. In immunocompromised patients, the intestinal parasites play a major role in causing chronic diarrhea accompanied by weight loss [15], with diarrhea reported in up to 50% of PLWHA in developed countries prior to the use of antiretroviral therapy (ART), and in up to 80% of those in resource-limited countries [14,16]. Antiretroviral therapy increases the length and quality of life and productivity of patients by improving survival and decreasing the incidence of opportunistic infections in PLWHA through the reduction of circulating viremia and increasing the level of CD4+ cells [16]. Previous studies in Nigeria have investigated intestinal parasitic infections in relation to ART and CD4+ count [17,18]. There is therefore the need for continuous surveillance of the prevalence and impact of these intestinal parasites among PLWHA, so as to provide guidance on prevention and control of co-infections, as well as reducing associated morbidity and mortality.

## Methods

**Study area:** This cross-sectional study was conducted at the HIV clinic of Federal Medical Centre, Abeokuta, Nigeria. This is one of the main treatment facilities for PLWHA in Abeokuta, Southern Nigeria.

**Ethics statement:** This study was approved by the Institutional Review Board of the Federal Medical Centre, Abeokuta, Nigeria. All participants gave informed consent before samples were collected and processed. General information regarding the nature of the study and its objectives was explained to participants, who were also informed of the right of refusal to participate in the study or to withdraw at any time, without jeopardizing their right of access to other health services. Identification numbers were used instead of names and information collected was kept confidential.

**Study population:** PLWHA positively confirmed patients, attending the HIV outpatient clinic of Federal Medical Centre, Abeokuta, Nigeria, who willingly gave informed consent and volunteered to have their stool samples examined were recruited into the study. Patients were attending clinic for routine check-up, collection of medications or other medical complaints. HIV status was confirmed using Determine HIV1/2 kit (Abbott Diagnostic Division, Hoofddorp, The Netherlands), followed by Unigold or Stat-Pak assay concurrently according to serial algorithm of the Federal Government of Nigeria. Socio-demographic data of the patients were collected.

**Sample collection and staining methods:** Fresh stool samples were collected in clean universal bottles labeled with each patient's details. Thin smears were made from all fecal samples and stained with Webe's Cromothrope stain and Kinyoun stain to detect the presence of microsporidia and *Cryptosporidium spp*respectively [19,20]. Blood samples were analyzed for CD4+ T cell estimates by flow cytometry. Patients with loose/ watery stool who also confirmed the passage of such stool for 2 to 3 times within the last 24 hours were considered to be having diarrhea.

**Data analysis:** Data were analyzed using SPSS version 16.0 (SPSS Inc. Chicago, IL, USA). Prevalence of enteric parasites among PLWHA was compared to CD4+ T cell count, presence or absence of diarrhea and the use of ART using Pearson's chi-square (χ^2^) test or Fisher's exact test, where appropriate. Statistical significance for all analyses was determined at 5% level of significance.

## Results

A total of 231 PLWHA were enrolled into the study. Their mean age was 31.81±11.40 years (range: 16-59 years; 135 [58.4%] females, 96 [41.6%] males). Majority (95.2%) had CD4+ cell count ≤200 cells/mm^3^. Eighty four (36.4%) harbored at least one of the following parasites; *Cryptosporidium spp*. (22.5%), *Microsporidium*spp (3.0%), *Ascaris (A) lumbricoides* (20.8%), hookworms (6.5%), *S. stercoralis* (4.3%), *T. trichiura* (5.6%) and Taenia spp. (5.6%). Table 1 shows the general characteristics of our study group and the distribution of intestinal parasites detected in stool samples. The distribution of opportunistic intestinal parasites with respect to CD4+ cell count is presented in Table 2. All the intestinal parasites seen in the study were more prevalent in PLWHA with CD4+ cell count ≤ 200 cells/mm^3^. *Cryptosporidium spp*. (80.8%), *Microsporidium* spp. (71.4%) and *S. stercoralis* (70.2%) were significantly higher in PLWHA with CD4+ count ≤ 200 cells/mm^3^ compared those with CD4+ count ≤ 200 cells/mm^3^. Table 3 shows the relationship between intestinal parasites and diarrhea in the study population. One hundred and twenty four (53.7%) of the PLWHA presented with diarrhea-related diseases like extremely watery faeces and cramping abdominal pains. *Cryptosporidium spp* oocyst was seen in 84.6% of PLWHA with diarrhea, which was statistically significant (p=0.006) when compared with those without diarrhea (15.4%). Table 4 shows the distribution of intestinal parasite species among PLWHA that are anti-retroviral therapy (ART)-naïve and those on ART. The naïve ART PLWHA were generally more infected with the intestinal parasites in comparison to those PLWHA already on ART. A strongly statistical significant difference was observed for *Cryptosporidium spp* (ART 26.9% vs Naïve ART 73.1%; p = 0.000) and *A. lumbricoides* (ART 16.7% vs Naïve ART 83.3%; p=0.001).

**Table 1:** General characteristics and distribution of the intestinal parasitic infections among the HIV/AIDS patients

Patient characteristics	Number N=231 (%)
**Number of subjects**	231
**Male: Female**	96: 135
**Mean age (years)**	31.81±11.40
**Mean CD4+ count (cells/mm^3^)**	194.3
**CD4+ count <200/mm^3^**	220 (95.2)
**CD4+ count >200/mm^3^**	11 (4.8)
**Stool positive for *Cryptosporidium spp***	52 (22.5)
**Stool positive for *Microsporidium* spp**	7 (3.0)
**Stool positive for *Strongyloides stercoralis***	10 (4.3)
**Stool positive for *Ascaris lumbricoides***	48 (20.8)
**Stool positive for Hookworm**	15 (6.5)
**Stool positive for *Trichuris trichiura***	13 (5.6)
**Stool positive for *Taenia* species**	13 (5.6)
**Number of HIV-positive individuals with minimum of 1 intestinal parasite**	84 (36.4)
**Study subjects with diarrhea**	124 (53.7%)

**Table 2:** Distribution of intestinal parasites with respect to CD4^+^ count

Parasite type	NumberPositive	Number (%) CD4^+^ ≤200 cells/mm^3^	Number (%) CD4^+^ ≥200 cells/mm^3^	*p*-value
***Cryptosporidium spp***	52	42 (80.8)	10 (19.2)	<0.0001*
***Microsporidium* spp**	7	5 (71.4)	2 (28.6)	0.038*
***Strongyloides stercoralis***	10	7 (70.0)	3 (30.0)	0.008*
***Ascaris lumbricoides***	48	44 (91.7)	4 (8.3)	0.246
**Hookworm**	15	15 (100.0)	0 (0.0)	1.000
***Trichuris trichuria***	13	13 (100.0)	0 (0.0)	1.000
***Taenia* spp**	13	13 (100.0)	0 (0.0)	1.000

* Significant p<0.05

**Table 3:** Distribution of intestinal parasites with respect to diarrhea in HIV-positive individuals

Parasite type	NumberPositive	Number (%) with diarrhea	Number (%) without diarrhea	*p*-value
***Cryptosporidium spp***	52	44 (84.6)	8 (15.4)	0.006*
***Microsporidium* spp**	7	6 (85.7)	1 (14.3)	0.679
***Strongyloides stercoralis***	10	7 (70.0)	3 (30.0)	1.000
***Ascaris lumbricoides***	48	37 (70.1)	11 (22.9)	0.221
**Hookworm**	15	11 (73.3)	4 (26.7)	1.000
***Trichuris trichuria***	13	10 (76.9)	3 (23.1)	0.759
***Taenia* spp**	13	9 (69.2)	4 (30.8)	1.000

* Significant p < 0.05

**Table 4:** Distribution of intestinal parasites with respect to ART status in HIV-positive individuals

		Number of positive cases (%)		
Parasite type	Positive cases	Individualson ART (N=15)	Naïve ART (N=216)	*p*-value
***Cryptosporidium spp***	52	14 (26.9)	38 (73.1)	0.000*
***Microsporidium* spp**	7	1 (14.3)	6 (85.7)	0.396
***Strongyloides stercoralis***	10	2 (20)	8 (80.0)	0.076
***Ascaris lumbricoides***	48	8 (16.7)	40 (83.3)	0.001*
**Hookworm**	15	3 (20.0)	12 (80.0)	0.028
***Trichuris trichuria***	13	2 (15.4)	11 (84.6)	0.181
***Taenia* spp**	13	2 (15.4)	11 (84.6)	0.181

* Significant p<0.05

## Discussion

Parasitic infections are major public health concern among PLWHA in developing countries, particularly in sub-Saharan Africa, with its high burden of HIV infections [21]. Down-regulation of the immune system, a hallmark of HIV infection, renders an individual extremely susceptible to numerous opportunistic parasitic infections [22]. Among such infections, gastrointestinal parasites are some of the leading causes of morbidity and mortality in, and are a universally recognized problem in PLWHA [21-23]. In this study, the overall prevalence of intestinal parasitic infection among PLWHA was found to be 36.4%. The result is almost similar to the findings from Ethiopia [8], Cameroon [23], and Saudi Arabia [24], but lower than those previously reported in Nigeria [18], India [25], Cameroon [26] and Lao PDR [27]. Such differing prevalence rates might be due to differences in geographical location, sensitivity of diagnostic techniques, immune status of study participants, environmental hygiene and possible increased awareness, amongst others. Several species of intestinal opportunistic parasites have been reported among PLWHA [16], with *Cryptosporidium* spp, *Microsporidium* spp and *S. stercoralis* the most commonly encountered in this study. *Cryptosporidium* spp, which was found to be responsible for diarrhea in 10-20% of PLWHA worldwide [28], has been reported among PLWHA in Nigeria [29], and more importantly in those with CD4+ T-cell counts of less than 200 cells/mm^3^ [18]. The prevalence of *Cryptosporidium spp* in this study was 22.5%, and lower than the findings from other studies (range 44-55%) [18,30,31]. The relatively lower prevalence of the parasites in this study might be due to difference in immunity, diarrheic status, environmental and personal hygiene of the study participants. This study further confirms previous reports that increased susceptibility of HIV-seropositive individuals to parasitic infections is associated with CD4+ T-cell counts less than 200 cells/mm3. At higher CD4+ T-cell count, spontaneous clearing of the parasites take place [18,32]. *Cryptosporidium* spp, *Strongyloides stercoralis* and *Microsporidia* spp were observed to be significantly associated with lower CD4+ T cell count. Cellular immunity is the major defense against intestinal parasitic infections. The reduction in CD4+ T cell count by the virus predisposes PLWHA to these opportunistic parasitic infections.

Microsporidiosis in PLWHA remain challenging and the prevalence reported in our study (3%) was lower than those reported in previous studies in Nigeria [10,33,34]. Most of the positive samples were identified among PLWHA with CD4+ count less than 200 cells/µl. Similar results were previously reported in other countries [5,35-37]. Additionally, most of the positive samples were identified in diarrheic stools (85.7%) and only 14.3% in formed stools. A possible reason for the low prevalence rates could be lack of molecular or immune-fluorescent techniques, which are more sensitive for diagnosis of *Microsporidium* spp. and could have benefited our study. Five helminths (*A. lumbricoides*, hookworm, *S. stercoralis, Taenia spp* and *T. trichiura*) were detected in this study. A very high prevalence of *A. lumbricoides* (20.8%) and hookworm (6.5%) was observed. Surprisingly, the prevalence of *S. stercoralis* was very low (4.3%) in our study population, which was expected to have the highest prevalence, being commonly implicated in immuno-compromised individuals. From this study, it is clear that there are no specific prevalence patterns of intestinal helminths among PLWHA. Distribution of parasites may depend on ecological variation and behavioral activities.

Limited evidence suggests that antiretroviral therapy (ART) reduces prevalence of helminth infections in PLWHA [30,38]. In this study, the prevalence of both helminth and protozoa intestinal infections was higher in ART naïve PLWHA compared to those already on ART. The prevalence of both *Cryptosporidium* spp and *A. lumbricoides* was significantly higher in ART naïve PLWHA compared to those on ART and the result is similar to the study conducted in Ethiopia [39]. It is commonly reported that the use of ART among PLWHA considerably reduces parasitic infections, especially protozoal infections such as cryptosporidiosis, although the underlying mechanisms remain unclear. Naturally, improved cellular immunity [16,40,41] and overall drug effect of both ART and cotrimoxazole preventive therapy (CTX-P), are the commonly given reasons for the reduction of parasite burden [41-43]. This study show that the prevalence of cryptosporidiosis was significantly higher in PLWHA that presented with diarrhea confirming this parasite as an important opportunistic protozoan causing diarrhea among these individuals in Nigeria [18,29]. Both *Cryptosporidium* spp. and *Microsporidium* spp. are capable of actively proliferating under reduced cellular defenses, leading to voluminous intractable diarrhea, severe loss of body fluid and dehydration [1].

## Conclusion

In conclusion, this study reveals a high prevalence of opportunistic parasitic infections, significantly correlated with diarrhea and low CD4+ T cell count among PLWHA in Nigeria. Additionally, reduced prevalence of these opportunistic infections was observed among PLWHA who are already on ART. In view of the high prevalence of cryptosporidiosis and other intestinal parasites and its significant association with diarrhea, prompt surveillance of intestinal parasitic infections utilizing molecular and immune-fluorescent techniques for stool examination and subsequent treatment is therefore recommended. Further study is recommended to understand the actual impact of ART on intestinal parasitic infections among PLWHA.

### What is known about this topic

- Opportunistic parasitic infections like *Cryptosporidium* and *Microsporidium* have emerged as important human infections with the advent of HIV/AIDS;
- Opportunistic parasitic infections are responsible for harmful health effects like chronic diarrhea, weight loss, and malnutrition, which has been associated with death among AIDS patients;
- Opportunistic parasitic infections have increasingly being associated with low CD4+ counts in HIV individuals.

### What this study adds

- This study adds to the growing data on the prevalence and impact of enteric pathogen among PLWHA that will be useful in the design of intervention policy;
- HIV individuals who are Anti-retroviral therapy (ART) naïve were shown to have higher burden of infection compared to HIV individuals who are on ART;
- Overall prevalence of the enteric pathogen is still high compared to a previous report, which suggests that much effort is needed to control these pathogens among PLWHA.
